# Psychogenic fever and postural tachycardia syndrome among school-aged children and adolescents with fever of unknown origin

**DOI:** 10.1186/s13030-022-00238-1

**Published:** 2022-03-29

**Authors:** Yuko Ishizaki, Yoshitoki Yanagimoto, Yuri Fujii, Mana Yamamoto, Kazunari Kaneko

**Affiliations:** Department of Pediatrics, Kansai Medical University, Hirakata, Osaka Japan

**Keywords:** Fever of unknown origin, Psychogenic fever, Postural tachycardia syndrome, School-aged children, Adolescent

## Abstract

**Background:**

Although fever is a common symptom in pediatric practice, its origin is often unknown in pediatric patients. Psychogenic fever is a stress-related, psychosomatic disease observed especially in young women. This study aimed to estimate the prevalence of psychogenic fever in pediatric patients with fever of unknown origin by surveying the medical records of school-aged children and adolescents.

**Methods:**

The study subjects included 47 patients aged 6–15 years who visited the Department of Pediatrics in Kansai Medical University Medical Center between January 2006 and December 2020 with fever of unknown origin. Data on age, sex, final estimated diagnosis, and comorbid psychosocial issues were collected from the medical records.

**Results:**

The study was composed of 47 patients, including 22 male and 25 female patients (male/female ratio, 1:1.36). The mean age was 10.1 (standard deviation, 2.4) years for boys and 11.6 (standard deviation, 2.7) years for girls (*p* = .047). The final estimated diagnoses were psychogenic fever, physical disorder, infection of unknown origin, and miscellaneous in 18, 12, 12, and 5 patients, respectively. The most common comorbidity in these pediatric patients with psychogenic fever was postural tachycardia syndrome.

**Conclusion:**

Psychogenic fever was a common cause of fever of unknown origin in pediatric patients, and postural tachycardia was prevalent among children with psychogenic fever. Enhanced sympathetic response to stress might play an important role in both psychogenic fever and postural tachycardia.

## Background

Infection is the most common cause of fever, which is a frequent symptom in pediatric practice [1]. Fever of unknown origin (FUO) is observed in some pediatric patients. In adults, FUO is defined as the presence of a well-documented fever for a period of 3 weeks without any identifiable etiology after a 1-week investigation [2]. The etiology of FUO in pediatric patients remains obscure, and the most frequent cause of FUO in pediatric cases is infections, followed by collagen vascular diseases and neoplasms [3]. A survey-based study of medical records conducted at an urban, tertiary care hospital in Washington, D.C., revealed that most pediatric inpatients with FUO were discharged without a diagnosis, followed closely by those diagnosed with an infection [4]. Conversely, psychogenic fever (PF) is a stress-related, psychosomatic disease observed more commonly in young women [5]. PF can present as a high core-body temperature of up to 41 °C or as a low-grade fever of 37 °C–38 °C during acute or chronic stress. The underlying mechanisms of PF are distinct from those of infection-induced fever and involve the central and sympathetic nervous systems [6]. In a review article of 62 reports on PF in Japan, with the highest number of cases occurred in 13-year-olds, and the male/female ratio was 1:1.19 [7].

These facts raise the question of the prevalence of PF as an etiology of FUO in pediatric populations. We therefore conducted a survey-based study using medical records to determine the rate of PF in school-aged children and adolescents with FUO.

## Methods

### Subjects and methods

In this retrospective study, the medical records of 47 patients with FUO aged 6–15 years who visited the Department of Pediatrics in Kansai Medical University Medical Center between January 2006 and December 2020 were reviewed; FUO was identified with an ICD-10 code of 50.9. Data on age, sex, final estimated diagnosis, and comorbidities were extracted from the medical records.

### Diagnostic categories

Patients were divided into four groups by the final estimated diagnosis: patients later diagnosed with a physical disorder, patients diagnosed with infection of unknown origin, patients considered to have PF, and miscellaneous, including those evaluated during one-time consultation with insufficient description for diagnosis.

### Ethics approvals

This study was approved by the Ethics Committee of the Kansai Medical University Medical Center (No. 2021293).

## Results

### Background characteristics of the patients

The study included 22 male and 25 female patients with FUO, a male/female ratio of 1:1.36. The mean age was 10.1 (standard deviation, 2.4) years for boys and 11.6 (standard deviation, 2.7) years for girls (*p* = .047).

### Final estimated diagnosis

Table 1 summarizes the final estimated diagnoses in the subjects. Briefly, the most frequent diagnosis was PF (*N* = 18), followed by a physical disorder later (*N* = 12), infection of unknown origin (*N* = 12), and miscellaneous (*N* = 5). Eighteen patients with primary FUO who received a final diagnosis of PF did not exhibit abnormal findings on various physical tests and had fever only under stressful conditions. For 12 patients who were later confirmed to have a physical disorder, the diagnoses were *Chlamydia* pneumonia, acute nephritis, *Mycoplasma* pneumonia, sinusitis, aseptic meningitis, autonomic symptoms after Guillain–Barré syndrome, virus-associated hemophagocytic syndrome, necrotizing lymphadenitis, acute pyelonephritis, idiopathic segmental anhidrosis, periodic fever, and pneumonia of unknown etiology. For patients with infection of unknown origin, although specific pathogenic agents and infectious foci could not be identified, the etiology was considered to be infectious based on the presence of leukocytosis, leukopenia, and/or elevated C-reactive protein levels, among others.

**Table 1:** Distribution of the final estimated diagnoses among patients with fever of unknown origin

Age	Total	Physical disorder	Infection of unknown origin	Psychogenic fever	Miscellaneous
6	3	3	0	0	0
7	2	0	1	0	1
8	7	1	3	3	0
9	5	3	1	1	0
10	2	0	0	0	2
11	6	2	2	1	1
12	3	0	0	3	0
13	10	2	3	5	0
14	7	1	2	3	1
15	2	0	0	2	0
Total	47	12	12	18	5

### Characteristics and comorbidities of patients with PF

Among the 18 patients who received a final diagnosis of PF, the median age was 13 years, and there were 4 male and 14 female patients. In this group, the most frequent comorbidity was orthostatic intolerance (*N* = 10), followed by child abuse, including Munchausen syndrome by proxy (*N* = 5), hyperventilation syndrome (*N* = 1), bronchial asthma (*N* = 1), and severe constipation (*N* = 1). Ten patients who were diagnosed with orthostatic intolerance included 3 male and 7 female patients, with ages ranging between 11 and 15 years; all ten patients exhibited orthostatic tachycardia without hypotension and showed persistent low-grade fever. Five patients with child abuse were aged between 8 and 14 years, and four of the five patients were female.

Table 2 shows the characteristics of patients with psychogenic fever and orthostatic tachycardia. In addition to fever, headache and fatigue were the main complaints, and the most common symptom of suspected orthostatic intolerance was lightheadedness. All patients had a low-grade fever of 37 °C–38 °C in the afternoon or evening. The standing test was conducted in the morning when they were afebrile.

**Table 2:** Characteristics of patients with psychogenic fever and orthostatic tachycardia

ID	Age	Sex	Chief complaints	Symptoms of suspected orthostatic intolerance	Diurnal variation in fever
1	13	Female	Fever, menstrual pain	Lightheadedness	Afternoon
2	14	Female	Fever	Lightheadedness	Afternoon or evening
3	15	Female	Fever, insomnia	Fatigue in the morning	Afternoon
4	12	Male	Fever, headache, heel pain	Fatigue in the morning	Afternoon or evening
5	12	Female	Fever, fatigue, nausea	Lightheadedness	Afternoon or evening
6	15	Female	Fever, headache	Lightheadedness	Afternoon or evening
7	11	Female	Fever, headache, lightheadedness	Lightheadedness	Afternoon or evening
8	13	Female	Fever, fatigue	Chronic fatigue	Afternoon
9	13	Male	Nausea, loss of appetite, headache, insomnia	Chronic headache	Afternoon or evening
10	13	Male	Fever	Chronic fatigue	Evening

## Discussion

### Prevalence of PF in pediatric patients with FUO

The present study aimed to estimate the prevalence of PF in pediatric patients with FUO by surveying medical records of school-aged children and adolescents with FUO who visited the pediatric outpatient department of a university hospital in an urban setting. We found that PF was a major cause of FUO in children. To the best of our knowledge, few studies have investigated the rate of PF among patients with FUO. In a study of adult patients with FUO who visited a Division of Collagen Diseases in a Department of Gastroenterology and Hematology over two and a half years, Sakuraba et al. [8] reported that 2 of the 40 patients were diagnosed with PF. Okada et al. [9] reported that 16 patients were diagnosed with PF in a study of 1229 patients who visited the outpatient department of pediatric psychosomatic disorder at a university hospital during the study course of 15 years. In a study investigating PF among children with FUO, 4 out of 7 teenagers with FUO were diagnosed with malingering fever/PF [10].

In the present study of 47 patients with FUO who visited the pediatric department of a university hospital during a study period of 15 years, the most common diagnosis was PF, with a rate of 38.3%. This finding is noteworthy, although the study was a preliminary survey. That the highest number of cases occurred in 13 year-olds in the present study subjects was in agreement with a review by Oka et al. [7]. However, the male/female ratio of pediatric patients with PF reported by Oka et al. was 1:1.19 [7], whereas Kaneda et al. [11] reported a male/female ratio of 1:2.2 in adolescents with PF. In contrast, we observed a very high male/female ratio of 1:3.5 in the present study. Although the underlying reason remains unknown, we speculate that the high comorbidity rate of postural tachycardia syndrome (POTS) might have partially accounted for the increased preponderance of female pediatric patients with PF in the current study.

### Comorbidities of patients with PF

The most common comorbidity was orthostatic intolerance, found in 10 patients. According to the diagnostic criteria of orthostatic dysregulation in children [12], all 10 patients were diagnosed with POTS, which is characterized by a heart rate increase of > 35 beats/min after standing from a sitting position or a maximum heart rate of > 115 beats/min in the absence of orthostatic hypotension. The prevalence of POTS in children is not known because screening of orthostatic intolerance is not routine [13]. However, Matsushima reported that 15% of pediatric patients with orthostatic symptoms were diagnosed as having POTS in survey of children and adolescents in Japan [14]. In comparison, the prevalence of POTS in the pediatric patients with PF of the current study is extremely high. This result indicates that there is a common mechanism between PF and POTS, and tachycardia and hyperthermia are based on a common cause. Enhanced sympathetic response has been proposed as a mechanism of POTS among patients with PF. Oka et al. [15] suggested that the mechanism of PF was not the same as that of infection-induced fever but involved the central and sympathetic nervous systems. Signs of inflammatory reaction are not detected in blood tests of patients with PF, whereas β3 adrenoreceptor-mediated non-shivering thermogenesis is considered to play an important role in the development of enhanced hyperthermic response in patients with PF. Often, subjects with β-adrenergic hyperresponsiveness show symptoms of POTS [15, 16]. Moreover, Lkhagvasuren et al. [17, 18] reported the presence of sympathetic hyperreactivity as a cardiovascular reaction when standing up in patients with PF and that the increase in heart rate after standing up was significantly higher in these patients than in healthy subjects. Both adolescent [17] and adult patients [18] with PF have a greater heart rate response to orthostatic stress and exhibit an increased prevalence of POTS compared with healthy subjects. The high comorbidity rate of POTS in patients with PF found in the present study is consistent with these studies. If enhanced sympathetic response leads to both fever and tachycardia, stabilizing sympathetic activity may be effective for addressing both PF and the symptoms of POTS.

In the present study, child abuse was identified in five patients with PF. Similarly, Okada et al. [9] reported that child abuse was observed in children with PF and that presentation with FUO enabled cooperation with medical and child welfare institutions and educational facilities. Therefore, cooperation of related organizations is important to ensure the safety of the child, even in cases of PF without a serious physical cause.

## Limitations

The current study has several limitations. First, the data were limited to the medical records of one hospital in an urban area. The results might differ between urban and rural areas as well as between university and community hospitals. Therefore, future studies based on multicenter collaboration should be considered to clarify the current issues related to PF in children.

## Conclusion

PF was a common cause of FUO in children, and POTS was prevalent among children with PF. Enhanced sympathetic response to stress might play an important role in both fever and tachycardia.

## Data Availability

Not applicable.

## Abbreviations

FUO: Fever of unknown origin
PF: Psychogenic fever
POTS: Postural tachycardia syndrome
